# Anti-Type I Interferon Autoantibodies in COVID-19 and Systemic Lupus Erythematosus: A Comparative Review

**DOI:** 10.3390/antib15030050

**Published:** 2026-06-17

**Authors:** Xin Rong Lim, Ryan Xuan Wei Teo, Rae Yi Xin Par, Bernard Pui Lam Leung

**Affiliations:** 1 Department of Rheumatology, Allergy and Immunology, Tan Tock Seng Hospital, Singapore 308433, Singaporebernard.leung@singaporetech.edu.sg (B.P.L.L.); 2 Health and Social Sciences, Singapore Institute of Technology, Singapore 828608, Singapore

**Keywords:** COVID-19, systemic lupus erythematous, interferon, autoantibodies

## Abstract

Type I interferons (IFN-I), including IFN-α, IFN-β, and IFN-ω, are central to antiviral defence and immune regulation. Autoantibodies targeting IFN-I (anti-IFN-I AAbs) have emerged as key pathogenic factors in severe coronavirus disease 2019 (COVID-19) and are detectable in systemic lupus erythematosus (SLE), a prototypic IFN-driven autoimmune disease. Here we compare the prevalence and clinical impact of anti-IFN-I autoantibodies (Aabs) in COVID-19 and SLE based on a structured review of 53 studies from 2014 to 2025 and highlight the clinical associations and therapeutic opportunities presented by these autoantibodies. In COVID-19, neutralising anti-IFN-α and/or anti-IFN-ω AAbs were consistently associated with severe disease and impaired antiviral responses, particularly in older male populations. In SLE, anti-IFN-α AAbs were variably detected; neutralising antibodies were associated with reduced interferon gene signatures in some cohorts but inconsistent correlations with disease activity. Therapeutically, anti-IFN-I AAbs in COVID-19 may inform risk stratification and early antiviral strategies, whereas in SLE, IFN-α blockade, including IFN-α kinoid vaccination, demonstrates modulation of IFN signatures but variable clinical benefit. Notably, these findings reveal an immunological paradox: the same neutralising mechanism that impairs antiviral defence in COVID-19 may attenuate chronic IFN-driven inflammation in SLE. Taken together, anti-IFN-I AAbs exert context-dependent effects: pathogenic in acute viral infection yet potentially modulatory in chronic IFN-driven autoimmunity. Prospective longitudinal studies are required to further clarify their translational utility and long-term clinical impact.

## 1. Introduction

Cytokines are small glycoproteins that function as key signalling molecules in the regulation of innate and adaptive immunity [1]. Among them, interferons (IFNs) play a central role in antiviral defence, antiproliferative activity, and tumour surveillance [2,3]. IFNs are broadly classified into type I and type II families, each signalling through distinct cell-surface receptors to coordinate immune responses [2]. Type I interferons (IFN-I), comprising IFN-α, IFN-β, IFN-ω and related subtypes, orchestrate antiviral immunity and modulate adaptive immune responses [3]. Dysregulation of IFN-I pathways is implicated in both infectious susceptibility and autoimmune pathogenesis.

Disruption of cytokine signalling can impair host defence and immune regulation. One important mechanism is the development of anti-cytokine autoantibodies (ACAAs), endogenous immunoglobulins that bind to cytokines and may alter their bioavailability, half-life, or biological activity. Neutralising ACAAs have been increasingly recognised as causes of acquired immunodeficiency [4,5], most notably anti-IFN-γ autoantibodies (AAbs), which predispose to disseminated non-tuberculous mycobacterial (NTM) and other infections [6,7,8].

More recently, attention has turned to autoantibodies targeting type I interferons, which have emerged as clinically significant mediators of both infectious susceptibility and immune dysregulation. Type I IFNs signal through the type I IFN receptor (IFNAR) to induce interferon-stimulated genes (ISGs) and establish an antiviral state [2,3]. Neutralising anti-IFN-I autoantibodies (anti-IFN-I AAbs) that block this signalling axis have been strongly associated with severe coronavirus disease 2019 (COVID-19), where impaired early IFN responses permit unchecked viral replication [9]. In contrast, SLE is characterised by chronic, dysregulated activation of the type I IFN pathway, which drives inflammation, autoreactive B-cell activation, and disease flares [10,11,12]. This persistent IFN signature has become a validated therapeutic target, as evidenced by the clinical efficacy of IFNAR blockade with anifrolumab [13]. Although anti-IFN-I AAbs have been detected in subsets of patients with SLE [14], their functional and clinical significance remains incompletely understood, raising the question of whether these autoantibodies may paradoxically attenuate IFN-driven autoimmune pathology while compromising antiviral host defence.

Thus, while both COVID-19 and SLE involve perturbations of the IFN-I pathway, the biological and clinical consequences of anti-IFN-I AAbs appear context-dependent and potentially divergent. A structured comparative synthesis across infection and autoimmunity has not been systematically undertaken. We therefore conducted a comparative review to evaluate the prevalence, functional characteristics, and clinical implications of anti-IFN-I AAbs in COVID-19 and SLE, with particular focus on neutralising activity, clinical outcomes, and therapeutic implications across these contrasting disease settings. This comparison is particularly warranted given the duality of IFN-I neutralisation across these disease contexts, with important implications for therapeutic targeting of the IFN axis.

## 2. Materials and Methods

This systematic review was conducted in accordance with the guidelines of Preferred Reporting Items for Systematic Reviews and Meta-Analyses (PRISMA) [15]; the completed PRISMA checklist is provided as Appendix A Table A1. The protocol was registered on PROSPERO with the reference number CRD420261398310.

### 2.1. Search Strategy

A structured literature search was conducted to identify studies evaluating anti-IFN-I AAbs in COVID-19 and SLE. Six electronic databases were searched: PubMed, SAGE Journals, Web of Science, Wiley Online Library, ScienceDirect, and Google Scholar. The search strategy utilises terms and conditions related to COVID-19, SLE, interferons, and autoantibodies. The primary search terms included: (“COVID-19” OR “2019-nCoV” OR “SARS-CoV-2” OR “Coronavirus Disease 2019” OR “Severe Acute Respiratory Syndrome Coronavirus 2” OR “SLE” OR “systemic lupus erythematosus” OR “lupus”) AND (“interferon” OR “IFN”) AND (“autoantibody” OR “autoantibodies”). All searches were completed on 31 July 2025. Results were limited to articles published between January 2014 and July 2025. The complete list of search strategies for all databases used is provided (see Appendix A Table A2).

### 2.2. Eligibility Criteria

Eligibility criteria were defined according to the Population, Exposure, Comparator, Outcomes, and Study design (PICOS) framework.

Population: Human subjects diagnosed with COVID-19, SLE, or both.

Exposure: Presence, detection, or functional assessment of anti-IFN-I AAbs.

Comparator: Studies with or without comparator groups, including healthy controls or disease controls, were eligible where applicable.

Outcomes: Clinical, immunological, or laboratory outcomes related to anti-IFN-I AAbs, including disease severity, immune dysregulation, interferon signalling, infection susceptibility, or therapeutic response.

Study design: Original research studies, including observational cohort, case–control, cross-sectional, and interventional studies published between January 2014 and July 2025.

Studies were excluded if they were review articles, editorials, commentaries, conference abstracts without full manuscripts, animal or in vitro studies, or reports with incomplete data or unavailable full texts in English.

### 2.3. Study Selection, Data Collection Process and Analysis

Two reviewers (T.X.W.R. and R.P.Y.X.) independently conducted the primary literature screening under the supervision of L.X.R. and L.P.L.B., who provided methodological oversight and performed additional verification of study eligibility and quality assessment. Study selection was carried out in three stages: title screening, abstract review, and full-text assessment. Eligible studies were subsequently categorised into COVID-19, SLE, or overlapping topics. Included studies were qualitatively evaluated independently by both reviewers. Disagreements were resolved through discussion between the reviewers and, when necessary, consultation with the supervising authors to achieve consensus.

### 2.4. Data Extraction and Quality Assessment

Data extraction was performed independently by two reviewers (T.X.W.R. and R.P.Y.X.) using a predefined and standardised data extraction form developed prior to study screening. Extracted variables included study characteristics (first author, year of publication, country, and study design), population characteristics (disease category, sample size, age and sex distribution), and methodological details, including the type of interferon autoantibody assay performed and whether functional neutralisation testing was conducted. Clinical and immunological outcomes were also collected, including the number and proportion of patients with circulating and/or neutralising anti-IFN-I AAbs, reported associations with disease severity or activity, and relevant clinical outcomes where available. Extracted data were cross-checked between reviewers to ensure accuracy and completeness, with discrepancies resolved through discussion and consensus with the supervising authors when required.

### 2.5. Risk of Bias Evaluation and Evidence Synthesis

A formal validated quality assessment tool, such as the Newcastle–Ottawa Scale or the Joanna Briggs Institute Critical Appraisal Checklist, was not applied in this review. Instead, quality assessment was performed as part of the data extraction process, in which both reviewers independently evaluated each included study for methodological rigour, completeness of reporting, and consistency of data. Discrepancies were resolved through discussion and consensus with the supervising authors. This approach was adopted given the heterogeneity of study designs included in this review, which encompassed cohort studies, case series, cross-sectional analyses, and clinical trials, making application of a single standardised quality assessment instrument impractical. This approach is aligned with the Cochrane risk-of-bias tool for randomization, intervention adherence, and outcome reporting, with evidence certainty assessed in a manner analogous to the Grades of Recommendation, Assessment, Development, and Evaluation (GRADE) framework. This limitation is acknowledged and discussed further in Section 5.

## 3. Results

**Figure 1:**
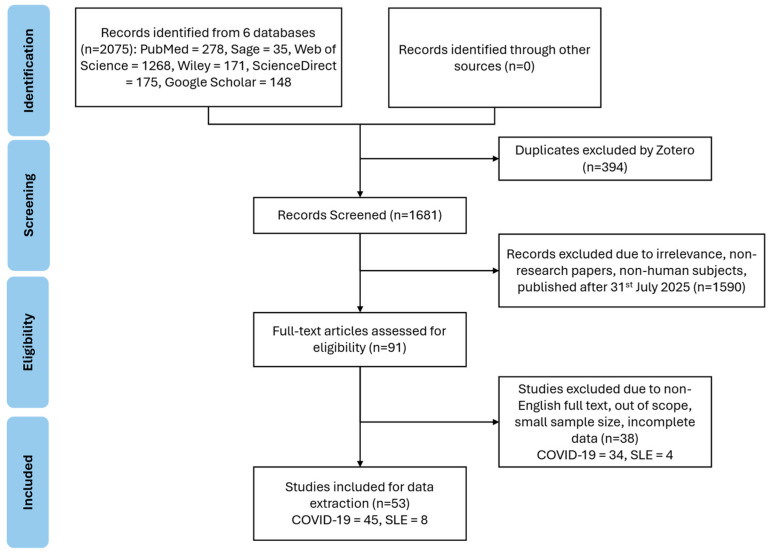
Flow diagram illustrating the identification, screening, eligibility, and inclusion of studies in this comparative review.

Following application of the search strategy and removal of duplicate records, a total of 1681 studies were identified. After title and abstract screening, 91 articles were selected for full-text review, of which 53 studies met the eligibility criteria and were included in the final data extraction (Figure 1).

The characteristics of the included studies involving SLE and COVID-19 are summarised in Appendix A Table A3. In addition, a comparative analysis was performed to evaluate the impact of anti-IFN-I AAbs in COVID-19 and SLE, focusing on population characteristics, clinical outcomes, and therapeutic implications (Table 1).

### 3.1. Prevalence of Anti-Type I IFN Autoantibodies

Multiple independent cohorts have demonstrated a substantial prevalence of neutralising anti-IFN-I AAbs among patients with severe COVID-19. Bastard et al. (2020) first identified neutralising IgG autoantibodies against type I IFNs in at least 10.2% (101/987) of patients with life-threatening COVID-19 pneumonia, but in none of 663 asymptomatic/mild cases and only 0.3% (4/1227) of healthy controls [9]. A subsequent 38-country study confirmed these neutralising anti-IFN-I AAbs in 13.6% (489/3595) of critical cases [16]. Comparable frequencies among critically ill patients have been reported across Europe and Asia: 7.9% in France (rising to 21% among deceased patients) [17], 9.5% in Barcelona [18], over 10% in Madrid [19], and 11.8% in Shanghai [20], with bronchoalveolar lavage confirming local neutralising activity in 13% of unvaccinated patients [21]. Lower but still significant frequencies of 4–5% were reported by Akbil et al. (2022) [22], Eto et al. (2022) [23], Covill et al. (2024) [24], and Lim et al. (2025) [25], again mainly in critically affected individuals. Paediatric rates were lower but detectable: 10% in severe COVID-19 [26], versus 0.5% and 4% in two other cohorts [27,28]. In the general population, these Aabs are uncommon (~1% at ages 20–70) but exceed 4% beyond age 70 [16].

In SLE, anti-IFN-I AAbs are present in approximately 3–15% of patients across most cohorts, with anti-IFN-α AAbs consistently the predominant subtype. The outlier reported by Harris et al. (2020) [29], who detected anti-IFN-α AAbs in 28 of 41 patients (68%), likely reflects assay sensitivity, study design, and small sample size rather than true disease population-level enrichment. Only a subset is neutralising; neutralising anti-IFN-α AAbs were typically found in approximately 3–6% of patients (Mathian et al. (2022, 5%) [30]; Bradford et al. (2023, 6%) [31]; Harris et al. (2020, 5%) [29].

### 3.2. Age and Sex Association

In SLE, only two studies specifically evaluated demographic associations between patients with and without anti-IFN-α AAbs. Both Beydon et al. (2022) [14] and Mathian et al. (2022) [30] reported no significant differences in age or sex distribution between seropositive and seronegative patients. In the cohort by Beydon et al., 17 of 20 anti-IFN-α–positive patients and 140 of 160 anti-IFN-α-negative patients were women, reflecting the expected female predominance of SLE but without enrichment among antibody-positive individuals. Similarly, no age differences were observed between groups in either study. Importantly, Mathian et al. further demonstrated that patients harbouring neutralising anti-IFN-α AAbs were distributed across similar age ranges, indicating that functional antibody status in SLE is not age-dependent.

In contrast, COVID-19 cohorts show a striking demographic skew. The prevalence of neutralising anti-type I IFN AAbs increases markedly with age, particularly after 70 years, rising from approximately 1% in individuals aged 20–70 years to over 4% in those older than 70 [16]. This age-dependent distribution likely contributes to the disproportionately high COVID-19 mortality observed in elderly populations. Among patients with neutralising anti-IFN-I AAbs, the proportion of males ranged from 78% to 100% across studies. Sex bias may partially explain the higher mortality rates observed among men with severe COVID-19.

### 3.3. Clinical Outcomes and Associations

Mortality and Severity in COVID-19:

Neutralising AAbs against type I interferons (IFN-I) are strongly associated with increased mortality and disease severity in COVID-19. Bastard et al. (2021) [16] first demonstrated that the prevalence of neutralising anti-IFN-I AAbs among patients with critical COVID-19 was substantially higher than in uninfected controls, with an age-dependent increase further amplifying risk in elderly populations. Subsequent work by Manry et al. (2022) [32] demonstrated that these AAbs significantly increase infection fatality rates across age groups and sexes.

These associations have been consistently replicated. Akbil et al. (2022) [22] reported markedly reduced survival in patients with neutralising IFN-I AAbs (7.7% vs. 80.9%), while Chauvineau-Grenier et al. (2021) found them in 21% of fatal cases [17].

Beyond mortality, these AAbs correlate with markers of immune dysregulation, including elevated C-reactive protein (CRP), D-dimer, and lymphopenia (Shi et al.) [20]. Emerging evidence also suggests that non-neutralising anti-IFN-I AAbs may contribute to thrombotic and cardiovascular complications (Framil et al., 2025) [33].

Secondary Infections:

In addition to influencing primary disease severity, anti-IFN-I AAbs predispose patients to secondary viral reactivations. Busnadiego et al. (2022) reported that 100% of COVID-19 patients with anti-IFN AAbs experienced herpesvirus reactivation, particularly cytomegalovirus (CMV), with a significantly elevated odds ratio (7.28) [34]. Detection of these AAbs in tracheobronchial secretions predicted viral reactivation, suggesting that local IFN pathway impairment contributes to susceptibility. Supporting this broader vulnerability to viral infections, Mathian et al. (2022) observed that SLE patients with neutralising anti-IFN-α AAbs had an increased risk of herpes zoster in addition to severe COVID-19 [30]. Beydon et al. (2022) reported a significant association between prior-tuberculosis and the presence of anti-IFN-α AAbs in SLE patients compared to those without such AAbs [14]. This observation raises the possibility that impaired type I IFN signalling may influence host responses beyond viral immunity, potentially contributing to altered susceptibility or disease course in certain bacterial infections.

Long COVID:

Three studies specifically assessed the association between anti-IFN-I AAbs and long COVID (post-acute sequelae of SARS-CoV-2 infection). Hansen et al. (2023) evaluated 279 patients experiencing long COVID symptoms and detected circulating anti-IFN-α AAbs in only 2% of cases, with no evidence of neutralising activity tested [35]. Similarly, Peluso et al. (2022) assessed 215 patients and identified anti-IFN-α AAbs in only 1% [36]. These findings suggest that anti-IFN-I AAbs do not play a major role in the pathogenesis of long COVID [37].

SLE disease activity:

Multiple studies demonstrated that neutralising anti-IFN-α AAbs were associated with lower SLE disease activity. Bradford et al. found these AAbs linked to inactive global disease scores and normalised B-cell subsets [31]. Mathian et al. (2022) confirmed that SLE patients with neutralising anti-IFN-α AAbs exhibited reduced serum IFN-α concentrations and lower disease activity [30]. Consistent with these findings, Gupta et al. (2016) demonstrated that SLE patients with blocking anti-IFN-α AAbs showed normalisation of the type I interferon gene expression signature [38].

In contrast, Beydon et al. (2022) found no significant difference in disease activity between anti-IFN-α Aab-positive and -negative patients [14]. The authors noted, however, that most patients in their cohort had low baseline disease activity, which may have limited the ability to detect differences based on SLE Disease Activity Index (SLEDAI) scores. Harris et al. (2020) reported that SLE serum samples containing anti-IFN-α AAbs reactive to three or more IFN-α subtypes exhibited significantly higher SLEDAI scores compared with samples binding two or fewer IFN-α subtypes [29]. Notably, neutralising activity was confirmed in only two samples, suggesting that broader binding reactivity rather than functional neutralisation may have been associated with increased disease activity in that cohort.

### 3.4. Therapeutic Interventions

Evidence for targeted therapeutic strategies in patients with anti-IFN-I AAbs is limited, but emerging data suggest potential benefit from immunomodulation. A German cohort found that therapeutic plasma exchange (TPE) reduced circulating levels of neutralising IFN AAbs and improved survival in critically ill COVID-19 patients, implying that antibody removal may partially restore antiviral immune function [22]. Chauvineau-Grenier et al. reported markedly lower mortality with tocilizumab in COVID-19 patients carrying neutralising anti-IFN-I AAbs (1/6 [17%] vs. 5/5 [100%] deaths; *p* = 0.01), suggesting that downstream cytokine blockade can mitigate hyper-inflammation despite impaired IFN-I signalling [39]. Although based on small numbers, these data support stratified immunomodulatory therapy in this high-risk subgroup.

Importantly, neutralising anti-IFN-α AAbs did not impair humoral responses to COVID-19 vaccination in SLE patients [30], and vaccination remained protective in autoimmune polyendocrine syndrome type I (APS-1) patients with neutralising anti-IFN-I AAbs [40], reinforcing current vaccination recommendations. Overall, while TPE and targeted cytokine inhibition may benefit selected patients, prospective trials are needed to define optimal management and whether systematic screening should guide therapy.

The development of IFN-α kinoid (IFN-K) underscores an immunological paradox. In severe COVID-19, spontaneous neutralising anti-type I IFN AAbs are pathogenic, impairing early antiviral defence; in SLE, therapeutic induction of neutralising anti-IFN-α antibodies through IFN-K appears beneficial by dampening the chronic IFN-I activation that drives lupus immunopathology [41,42,43]. Thus, IFN-α neutralisationcan be deleterious or therapeutic depending on disease context, reflecting the dual role of type I IFNs and the importance of timing and immune milieu. Understanding this balance is critical for precision approaches targeting the IFN axis across infectious and autoimmune disease.

## 4. Discussion

### 4.1. Summary of Evidence

This review synthesises 53 studies of anti-IFN-I AAbs in COVID-19 and SLE, encompassing more than 14,000 patients across multiple continents. Collectively, the data establish these autoantibodies as a major determinant of severe COVID-19 outcomes while revealing a paradoxical immunomodulatory role in SLE.

Neutralising anti-IFN-I AAbs were detected in approximately 4–38% of severe or critical COVID-19 cases depending on the cohort and assay methodology and accounted for a substantial proportion of COVID-19-related deaths. Their prevalence is strongly age-dependent, increasing from approximately 1% among individuals aged 20–70 years to over 4% in those older than 70 years [16], with lower but clinically relevant frequencies in children [26,27,28]. In SLE, neutralising anti-IFN-α AAbs are present in approximately 3–6% of cases and are associated with lower lupus disease activity but increased susceptibility to severe viral infections. Beyond neutralising AAbs, non-neutralising binding anti-IFN-I AAbs have been reported in approximately 2–53% of COVID-19 patients and 3–68% of SLE patients depending on assay platform and cohort (Appendix A Table A3), though their clinical significance remains unclear.

This variability in prevalence estimates for neutralising and binding autoantibodies likely reflects interstudy heterogeneity in assay methodology. Binding assays detect serological recognition of type I interferons (IFN-I) but not functional impairment, whereas neutralising assays confirm blockade of IFN-I signalling. Binding-only studies generally reported higher prevalence estimates; for example, Harris et al. (2020) [29] reported 68% binding positivity but only 5% neutralising activity. Differences in IFN subtype assessed, assay thresholds, and positivity criteria further limit comparability across cohorts. As shown in Appendix A Table A3, most COVID-19 studies incorporated neutralisation testing, whereas such neutralising data were available in only four of eight SLE studies [29,30,31,38]. These methodological inconsistencies remain a major barrier to clinical translation, particularly for risk stratification.

Beyond COVID-19 severity, anti-IFN-I AAbs may increase susceptibility to secondary infections, particularly herpesvirus reactivation [34], by neutralising circulating IFN-I and blocking downstream IFNAR signalling. Non-neutralising anti-IFN-I AAbs may also be clinically relevant, with reported associations with thrombotic and cardiovascular complications [33], consistent with broader links between antiphospholipid antibodies and thrombosis in COVID-19 [44].

### 4.2. The Interferon Paradox: Infection Risk Versus Autoimmune Control

**Figure 2:**
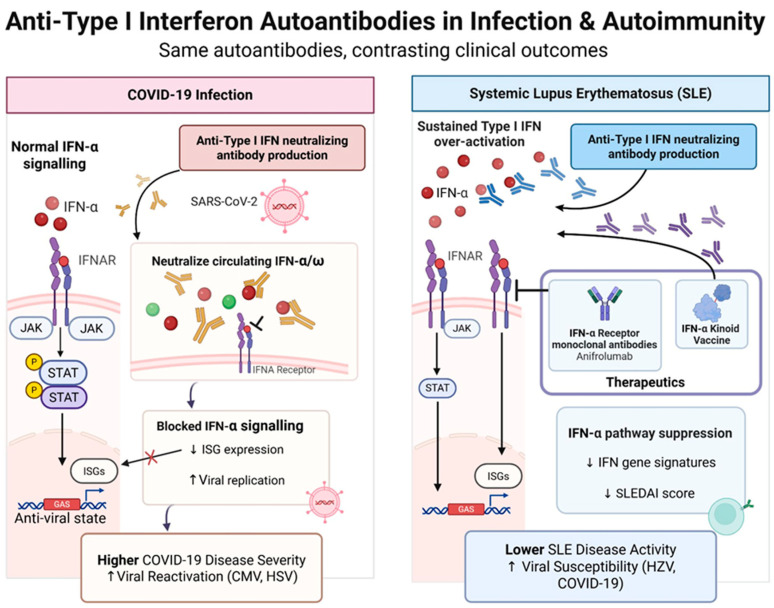
Context-dependent effects of anti-IFN-I AAbs in viral infection and SLE. In viral infection, neutralising anti-IFN-I AAbs block IFN-α/ω binding to IFNAR, impairing JAK1–STAT1/STAT2 signalling and ISG induction, resulting in diminished antiviral responses, increased viral replication, and heightened susceptibility to viral reactivation. In SLE, where chronic overactivation of the type I IFN pathway drives inflammation and disease activity, anti-IFN-I AAbs and therapeutic IFN pathway inhibitors (e.g., anifrolumab, IFN-α kinoid vaccines) partially suppress IFN signalling, reducing ISG expression and disease activity but potentially increasing susceptibility to viral infections. Created in BioRender. Teo, R. (2026) https://BioRender.com/jwqxo79 (accessed on 7 April 2026). Antibody icons represent neutralising anti-type I IFN autoantibodies; coloured circles represent IFN-α cytokines. Abbreviations: IFN, interferon; IFNAR, interferon-α receptor (type I IFN receptor); JAK1, Janus kinase 1; STAT, signal transducer and activator of transcription; ISG, interferon-stimulated gene; SLEDAI, SLE Disease Activity Index; CMV, cytomegalovirus; HSV, herpes simplex virus; GAS, gamma-activated sequence.

These findings illuminate a fundamental immunological paradox: the same neutralising autoantibodies that predispose to life-threatening viral infections appear to attenuate autoimmune inflammation in SLE (Figure 2). This duality reflects the central role of type I IFNs as both indispensable antiviral mediators and potent amplifiers of autoimmune pathology.

In SLE, chronic type I IFN pathway activation promotes autoreactive B-cell activation, plasma cell differentiation, dendritic cell maturation, and pro-inflammatory cytokine production. Neutralising anti-IFN-α AAbs attenuate this pathway and are associated with lower IFN activity, reduced IFN gene signature expression, decreased disease activity, and partial normalisation of immune abnormalities.

Anifrolumab, a monoclonal antibody directed against the type I IFN receptor subunit 1 (IFNAR1) that inhibits signalling from all type I IFNs, has demonstrated clinical efficacy in moderate-to-severe SLE [45]. However, it remains uncertain whether patients with pre-existing neutralising anti-IFN-α AAbs derive similar benefit, particularly given their lower baseline IFN gene signature. Stratified analyses are needed to determine whether baseline anti-IFN-α AAb status modifies treatment response.

Attenuation of IFN signalling comes at a cost. SLE patients with neutralising anti-IFN-α AAbs demonstrate markedly increased risk of severe COVID-19 and herpes zoster, highlighting impaired antiviral defence. This highlights the balance between disease control and infection risk in IFN-targeted therapy.

### 4.3. Risk Stratification and Clinical Management

The robust association between anti-IFN-I AAbs and severe COVID-19 suggests potential utility in risk stratification. Early detection in COVID-19: Testing for neutralising anti-IFN-I AAbs in patients with severe disease, advanced age, or other risk factors may enable enhanced monitoring and early therapeutic intervention. Identification of this immunological endotype could guide enrolment into targeted treatment protocols or clinical trials.

Implications for SLE management: In SLE populations, the presence of neutralising anti-IFN-α AAbs should prompt heightened vigilance for infections, particularly viral reactivation. Aggressive vaccination strategies, including COVID-19 and herpes zoster vaccination, remain strongly supported, as vaccine immunogenicity does not appear to be impaired by these AAbs. Clinicians may also consider infection risk when tailoring immunosuppressive regimens.

Population screening considerations: The relatively higher prevalence of anti-IFN-I AAbs among individuals over 70 years old and their strong association with COVID-19 mortality raise the question of targeted screening in high-risk groups. However, the cost-effectiveness, feasibility, and impact on clinical outcomes remain to be determined.

## 5. Limitations

### 5.1. Study-Level Limitations

Substantial variability existed across studies in assay platforms, interferon subtype targets, and definitions of neutralising activity. Methods ranged from ELISA and multiplex bead arrays to luciferase reporter-based neutralisation assays, Gyros immunoassays, radioligand binding assays, and cell-based functional systems. Differences in assay sensitivity, specificity, and threshold definitions complicate direct comparisons and preclude quantitative pooling. Importantly, not all studies distinguished clearly between binding and functional neutralising antibodies, which may have differential clinical relevance. Most COVID-19 studies preferentially enrolled hospitalised patients with severe or critical disease, potentially inflating estimates of prevalence and clinical impact. Mild and asymptomatic infections were comparatively underrepresented, limiting inference regarding the broader infected population.

In contrast, SLE cohorts were often derived from tertiary referral centres, which may overrepresent patients with more complex disease phenotypes. The majority of studies assessed anti-IFN-I AAbs at a single time point. Consequently, limited data exist regarding longitudinal dynamics, persistence after infection, induction versus pre-existence, or changes with immunomodulatory treatment. Whether these antibodies represent stable immunological traits or context-dependent phenomena remains incompletely defined. Compared with COVID-19, relatively fewer studies have evaluated anti-IFN-I AAbs in SLE, and sample sizes were generally smaller. Functional neutralisation testing was inconsistently performed. As a result, conclusions regarding prevalence, disease-modifying effects, and infection susceptibility in SLE remain less definitive.

### 5.2. Review-Level Limitations

Given the marked heterogeneity in study populations, assay methodologies, and reported outcomes, a quantitative meta-analysis was not feasible. The synthesis was therefore narrative, limiting the precision of pooled prevalence and effect size estimates. The included literature was predominantly published in English-language journals, often with positive findings. In addition, formal validated quality assessment tools, such as the Cochrane Risk of Bias 2 and GRADE framework, were not applied; instead, both reviewers independently evaluated each study for methodological rigour during data extraction, with discrepancies resolved by consensus with the supervising authors.

## 6. Future Perspectives

Several priority areas warrant investigation to advance the clinical translation of anti-IFN-I AAb research. First, the development of standardised, validated assays that reliably distinguish binding from neutralising AAbs is essential to enable cross-study comparisons and inform screening strategies. Second, prospective longitudinal studies are needed to determine whether anti-IFN-I AAbs represent stable immunological traits or dynamic markers influenced by disease state, treatment, and ageing. Third, the interaction between pre-existing anti-IFN-I AAbs and response to IFN-targeted therapies such as anifrolumab in SLE requires clarification through stratified analyses in clinical trials and real-world cohorts. Fourth, the potential role of anti-IFN-I AAbs in risk stratification for severe outcomes in future pandemics and respiratory viral infections should be evaluated in population-based screening studies. Finally, investigation of the relationship between anti-IFN-I AAbs and other immune-mediated prothrombotic mechanisms, including antiphospholipid antibodies, may yield insights into the broader immunopathology of severe COVID-19 and inform therapeutic approaches.

## 7. Conclusions

Anti-IFN-I AAbs play a context-dependent role in infection and autoimmunity. In COVID-19, neutralising anti-IFN-I AAbs are present in approximately 10% of patients with severe disease, account for a substantial proportion of COVID-19 deaths, and are strongly associated with advanced age and male sex. They define a high-risk immunological endotype characterised by impaired antiviral defence.

In contrast, in SLE, neutralising anti-IFN-α AAbs occur in a smaller subset of patients and are associated with reduced IFN pathway activity and, in several cohorts, lower disease activity. However, this attenuation of IFN signalling may increase susceptibility to viral infections, illustrating the dual role of type I IFNs in human immunity.

These findings highlight the “interferon paradox”: IFN neutralisation may be pathogenic in acute viral infection, yet modulatory in chronic autoimmune disease. Standardised detection methods, longitudinal studies, and targeted clinical trials are needed to clarify their role in risk stratification and precision therapeutic strategies across both settings.

## Figures and Tables

**Table 1:** Comparison of Anti-Type I IFN Autoantibodies in COVID-19 and SLE.

Domain	COVID-19	SLE
Number of Studies Included	45	8
Population Characteristics	• More frequently detected in males and individuals of advanced age. • Detected in paediatric populations at lower frequencies, with variable clinical significance.	• No consistent association with age or sex.
Clinical Impact of Anti-Type I IFN Autoantibodies	• Associated with reduced circulating IFN activity and impaired antiviral responses. • Strongly associated with severe and critical COVID-19. • Associated with increased mortality in multiple cohorts. • No clear association with long COVID.	• Elevated IFN-α levels correlate with lupus disease activity. • Neutralising anti-IFN-α autoantibodies associated with reduced IFN gene signature and lower SLE disease activity in some cohorts. • Increased susceptibility to severe viral infections (e.g., COVID-19, herpes zoster). • Relationship with bacterial infection susceptibility (e.g., tuberculosis) requires further investigation.
Therapeutic Implications	• Early identification may support risk stratification and intensified monitoring. • May benefit from early antiviral or immunomodulatory therapy. • Vaccination (including boosters) strongly recommended. • Experimental approaches include plasma exchange and targeted cytokine blockade.	• IFNAR inhibition (e.g., anifrolumab) demonstrates clinical benefit. • IFN-α kinoid (IFN-K) induces neutralising anti-IFN-α antibodies and reduces IFN gene signature. • Infection vigilance and vaccination (COVID-19, herpes zoster) are essential.

Abbreviations: COVID-19, coronavirus disease 2019; IFN, interferon; SLE, systemic lupus erythematosus; IFNAR, interferon alpha receptor.

## Data Availability

The data presented in this study are available on request from the corresponding author.

## Abbreviations

The following abbreviations are used in this manuscript:

Abbreviation	Full Term
AAbs	Autoantibodies
ACAAs	Anti-cytokine autoantibodies
ALBIA	Addressable laser bead immunoassay
APS-1	Autoimmune polyendocrine syndrome type 1
BAL	Bronchoalveolar lavage
CMV	Cytomegalovirus
COVID-19	Coronavirus disease 2019
ELISA	Enzyme-linked immunosorbent assay
HSV	Herpes simplex virus
HLA	Human leukocyte antigen
IFN	Interferon
IFN-I	Type I interferon
IFN-α	Interferon alpha
IFN-β	Interferon beta
IFN-ω	Interferon omega
IFNAR	Interferon alpha receptor
IFN-K	Interferon alpha kinoid
ISG	Interferon-stimulated gene
JAK1	Janus kinase 1
LIPS	Luciferase immunoprecipitation system
NR	Not reported
NTM	Non-tuberculous mycobacteria
PICOS	Population, Intervention, Comparator, Outcomes, Study design
RLBA	Radioligand binding assay
SARS-CoV-2	Severe acute respiratory syndrome coronavirus 2
SLE	Systemic lupus erythematosus
SLEDAI	Systemic Lupus Erythematosus Disease Activity Index
STAT	Signal transducer and activator of transcription
TPE	Therapeutic plasma exchange

## Appendix A

**Table A1:** PRISMA 2020 Abstracts and Main Checklist.

**Section and Topic**	**Item #**	**Checklist Item**	**Reported (Yes/No)**
Title			
Title	1	Identify the report as a systematic review.	No, as a comparative review
Background			
Objectives	2	Provide an explicit statement of the main objective(s) or question(s) the review addresses.	Yes
Methods			
Eligibility criteria	3	Specify the inclusion and exclusion criteria for the review.	Yes
Information sources	4	Specify the information sources (e.g., databases, registers) used to identify studies and the date when each was last searched.	Yes
Risk of bias	5	Specify the methods used to assess risk of bias in the included studies.	Yes
Synthesis of results	6	Specify the methods used to present and synthesise results.	Yes
Results			
Included studies	7	Give the total number of included studies and participants and summarise relevant characteristics of studies.	Yes
Synthesis of results	8	Present results for main outcomes, preferably indicating the number of included studies and participants for each. If meta-analysis was done, report the summary estimate and confidence/credible interval. If comparing groups, indicate the direction of the effect (i.e., which group is favoured).	Yes
Discussion			
Limitations of evidence	9	Provide a brief summary of the limitations of the evidence included in the review (e.g., study risk of bias, inconsistency and imprecision).	Yes
Interpretation	10	Provide a general interpretation of the results and important implications.	Yes
Other			
Funding	11	Specify the primary source of funding for the review.	No This research received no external funding.
Registration	12	Provide the register name and registration number.	Yes
**Section and Topic**	**Item #**	**Checklist Item**	**Location Where Item Is Reported.**
Title			
Title	1	Identify the report as a systematic review.	Identified as a comparative review in title.
Abstract			
Abstract	2	See the PRISMA 2020 for Abstracts checklist.	Checklist attached
Introduction			
Rationale	3	Describe the rationale for the review in the context of existing knowledge.	Introduction, Paragraphs 1–4
Objectives	4	Provide an explicit statement of the objective(s) or question(s) the review addresses.	Introduction, Final paragraph
Methods			
Eligibility criteria	5	Specify the inclusion and exclusion criteria for the review and how studies were grouped for the syntheses.	Methods → Inclusion criteria/Exclusion criteria, Pages 2–3
Information sources	6	Specify all databases, registers, websites, organisations, reference lists and other sources searched or consulted to identify studies. Specify the date when each source was last searched or consulted.	Methods → Search strategy, Last search (31 July 2025)
Search strategy	7	Present the full search strategies for all databases, registers and websites, including any filters and limits used.	Appendix A (Full search strategies for all databases)
Selection process	8	Specify the methods used to decide whether a study met the inclusion criteria of the review, including how many reviewers screened each record and each report retrieved, whether they worked independently, and if applicable, details of automation tools used in the process.	Methods → Study selection
Data collection process	9	Specify the methods used to collect data from reports, including how many reviewers collected data from each report, whether they worked independently, any processes for obtaining or confirming data from study investigators, and if applicable, details of automation tools used in the process.	Methods → Data extraction
Data items	10a	List and define all outcomes for which data were sought. Specify whether all results that were compatible with each outcome domain in each study were sought (e.g., for all measures, time points, analyses), and if not, the methods used to decide which results to collect.	Methods → Inclusion criteria (Primary outcomes) Table A3
	10b	List and define all other variables for which data were sought (e.g., participant and intervention characteristics, funding sources). Describe any assumptions made about any missing or unclear information.	Methods → Data extraction Table A2 and Table A3
Study risk of bias assessment	11	Specify the methods used to assess risk of bias in the included studies, including details of the tool(s) used, how many reviewers assessed each study and whether they worked independently, and if applicable, details of automation tools used in the process.	Methods → Risk of Bias Assessment
Effect measures	12	Specify for each outcome the effect measure(s) (e.g., risk ratio, mean difference) used in the synthesis or presentation of results.	Methods → Data extraction.
Synthesis methods	13a	Describe the processes used to decide which studies were eligible for each synthesis (e.g., tabulating the study intervention characteristics and comparing against the planned groups for each synthesis (item #5)).	Methods → Section 2.2, Section 2.3 and Section 2.4
	13b	Describe any methods required to prepare the data for presentation or synthesis, such as handling of missing summary statistics, or data conversions.	Methods → Data extraction
	13c	Describe any methods used to tabulate or visually display results of individual studies and syntheses.	Table A2 and Table A3 Figure 1
	13d	Describe any methods used to synthesise results and provide a rationale for the choice(s). If meta-analysis was performed, describe the model(s), method(s) to identify the presence and extent of statistical heterogeneity, and software package(s) used.	Under Results section
	13e	Describe any methods used to explore possible causes of heterogeneity among study results (e.g., subgroup analysis, meta-regression).	Not formally analysed; discussed narratively.
	13f	Describe any sensitivity analyses conducted to assess robustness of the synthesised results.	No sensitivity analyses were conducted due to the small number of included studies; this is stated in the Discussion section.
Reporting bias assessment	14	Describe any methods used to assess risk of bias due to missing results in a synthesis (arising from reporting biases).	Described under study-level and review level limitations.
Certainty assessment	15	Describe any methods used to assess certainty (or confidence) in the body of evidence for an outcome.	Methods → quality assessment was performed as part of the data extraction process, in which both reviewers independently evaluated each included study for methodological rigour, completeness of reporting, and consistency of data.
Results			
Study selection	16a	Describe the results of the search and selection process, from the number of records identified in the search to the number of studies included in the review, ideally using a flow diagram.	Results → first paragraph, and Figure 1 (PRISMA Flow Chart)
	16b	Cite studies that might appear to meet the inclusion criteria, but which were excluded, and explain why they were excluded.	Results → first paragraph, and Figure 1
Study characteristics	17	Cite each included study and present its characteristics.	Results, and Table 1 and Table A3
Risk of bias in studies	18	Present assessments of risk of bias for each included study.	Results → As a comparative review, we did not perform risk of bias in all individual study.
Results of individual studies	19	For all outcomes, present, for each study: (a) summary statistics for each group (where appropriate) and (b) an effect estimate and its precision (e.g., confidence/credible interval), ideally using structured tables or plots.	Discussion, Section 4.1
Results of syntheses	20a	For each synthesis, briefly summarise the characteristics and risk of bias among contributing studies.	Results → Section 3.1, Section 3.2, Section 3.3 and Section 3.4
	20b	Present results of all statistical syntheses conducted. If meta-analysis was done, present for each the summary estimate and its precision (e.g., confidence/credible interval) and measures of statistical heterogeneity. If comparing groups, describe the direction of the effect.	None as this is not a meta-analysis.
	20c	Present results of all investigations of possible causes of heterogeneity among study results.	Not formally analysed; discussed narratively.
	20d	Present results of all sensitivity analyses conducted to assess the robustness of the synthesised results.	Not conducted; stated in Discussion
Reporting biases	21	Present assessments of risk of bias due to missing results (arising from reporting biases) for each synthesis assessed.	Results sections and limitations under discussion
Certainty of evidence	22	Present assessments of certainty (or confidence) in the body of evidence for each outcome assessed.	Results sections and limitations under discussion
Discussion			
Discussion	23a	Provide a general interpretation of the results in the context of other evidence.	Discussion → first 3 paragraphs
	23b	Discuss any limitations of the evidence included in the review.	Discussion and Limitations
	23c	Discuss any limitations of the review processes used.	Discussion and Limitations
	23d	Discuss implications of the results for practice, policy, and future research.	Discussion → final paragraphs
Other Information			
Registration and protocol	24a	Provide registration information for the review, including register name and registration number, or state that the review was not registered.	
	24b	Indicate where the review protocol can be accessed, or state that a protocol was not prepared.	Methods → Protocol and registration
	24c	Describe and explain any amendments to information provided at registration or in the protocol.	Same section PROSPERO, CRD420261398310 No amendments
Support	25	Describe sources of financial or non-financial support for the review, and the role of the funders or sponsors in the review.	Funding
Competing interests	26	Declare any competing interests of review authors.	Conflicts of interest section
Availability of data, code and other materials	27	Report which of the following are publicly available and where they can be found: template data collection forms; data extracted from included studies; data used for all analyses; analytic code; any other materials used in the review.	Data Availability Statement

From: Page, M.J.; McKenzie, J.E.; Bossuyt, P.M.; Boutron, I.; Hoffmann, T.C.; Mulrow, C.D.; Shamseer, L.; Tetzlaff, J.M.; Akl, E.A.; Brennan, S.E.; et al. The PRISMA 2020 statement: An updated guideline for reporting systematic reviews. BMJ 2021, 372, n71. https://doi.org/10.1136/bmj.n71 [15]. This work is licenced under CC BY 4.0. To view a copy of this licence, visit https://creativecommons.org/licenses/by/4.0/.

**Table A2:** Literature Search Strategy.

No.	Databases	Search Terms	Results Total =
1	Pubmed	“Interferons” [MeSH Terms] AND “Autoantibodies” [MeSH Terms] AND (“COVID-19” [Mesh] OR “Lupus Erythematosus, Systemic” [Mesh]) Filters: From 2014–July 2025	278
2	Sage	In All Content: “COVID-19” OR “2019-nCoV” OR “SARS-CoV-2” OR “2019 Novel Coronavirus” OR “Coronavirus Disease 2019” OR “Severe Acute Respiratory Syndrome Coronavirus 2” OR “SLE” OR “Systemic Lupus Erythematosus” OR “lupus” In Title: (Interferon OR IFN) AND (autoantibod* OR auto-antibod*) Filter: Published Date from January 2014–July 2025	5
		In All Content: “COVID-19” OR “2019-nCoV” OR “SARS-CoV-2” OR “2019 Novel Coronavirus” OR “Coronavirus Disease 2019” OR “Severe Acute Respiratory Syndrome Coronavirus 2” OR “SLE” OR “Systemic Lupus Erythematosus” OR “lupus” In Abstract: (Interferon OR IFN) AND (autoantibod* OR auto-antibod*) Filter: Published Date from January 2014–July 2025	30
3	Web of Science	((COVID-19 (All Fields) or 2019-nCoV (All Fields) or SARS-CoV-2 (All Fields) or 2019 Novel Coronavirus (All Fields) or Coronavirus Disease 2019 (All Fields) or Severe Acute Respiratory Syndrome Coronavirus 2 (All Fields) or SLE (All Fields) or Systemic Lupus Erythematosus (All Fields) or Lupus (All Fields)) AND ((interferon (All Fields) or IFN (All Fields)) AND ((autoantibody (All Fields) or autoantibodies (All Fields)) Filter: Publication Date from 1 January 2014–31 July 2025	1268
4	Wiley	(Interferon OR IFN) AND (autoantibod* OR auto-antibod*) in Abstract and “COVID-19” OR “2019-nCoV” OR “SARS-CoV-2” OR “2019 Novel Coronavirus” OR “Coronavirus Disease 2019” OR “Severe Acute Respiratory Syndrome Coronavirus 2” OR “SLE” OR “Systemic Lupus Erythematosus” OR “lupus” anywhere Filter: Published Date from January 2014–July 2025	154
		(Interferon OR IFN) AND (autoantibod* OR auto-antibod*) in Title and “COVID-19” OR “2019-nCoV” OR “SARS-CoV-2” OR “2019 Novel Coronavirus” OR “Coronavirus Disease 2019” OR “Severe Acute Respiratory Syndrome Coronavirus 2” OR “SLE” OR “Systemic Lupus Erythematosus” OR “lupus” anywhere Filter: Published Date from January 2014–July 2025	17
5	Science Direct	(“COVID-19” OR “2019-nCoV” OR “SARS-CoV-2” OR “2019 Novel Coronavirus” OR “Coronavirus Disease 2019”) AND TITLE-ABS-KEY ((Interferon OR IFN) AND (autoantibody OR autoantibody)) Filters: 2014–2025	55
		(“SLE” OR “Systemic Lupus Erythematosus” OR “lupus”) AND TITLE-ABS-KEY ((Interferon OR IFN OR Interleukin OR IL) AND (autoantibody OR autoantibody)) Filters: 2014–2025	120
6	Google Scholar	Where my words occur: In the title of the article With the exact phrase: Autoantibody With at least one of the words: Interferon IFN Return articles dated between 2014 and 2025	148

The truncation symbol (*) was used to capture variations of a word stem (e.g., autoantibody, autoantibodies).

**Table A3:** Characteristics of the Included COVID-19 and SLE studies.

Author (Year of Publication)	Country	Type of Disease	% of Patients with Severe-Critical COVID-19 Illness	Biological Samples	Autoantibody Tested	Laboratory Assay	Tested for Neutralazing Capacity?	No. of Total Study Patients (% Male)	No. of Patients (% of Total) with Circulating Anti-IFN-1	No. of Patients (% of Total) with Neutralising Anti-IFN-1	No. of Male Patients (%) with Neutralising Anti-IFN-1	Breakdown of Circulating Anti-IFN-α/β/ω	Breakdown of Neutralising Anti-IFN-α/β/ω
Beydon et al. (2022) [14]	France	SLE	NR	Blood	aIFN-α	ELISA	N	180 (13%)	20 (11%)	-	-	20/-/-	-
Bondet et al. (2021) [46]	France/UK	SLE	NR	Blood	aIFN-α	LIPS	N	206 (8%)	24 (12%)	-	-	24/-/-	-
Bradford et al. (2023) [31]	UK/Estonia	SLE	NR	Blood	aIFN-α/β/ω	LIPS	Y	501 (8%)	75 (15%)	28 (6%)	2 (7%)	66/5/59	28/-/-
Choi et al. (2022) [47]	Canada	SLE	NR	Blood	aIFN-α/β	(ALBIA)/Luminex multiplex	N	173 (5%)	6 (3%)	-	-	6/0/-	-
Gan et al. (2014) [48]	US	SLE	NR	Blood	aIFN-α	LIPS	N	9 (NR)	1 (11%)	-		1/-/-	-
Gupta et al. (2016) [38]	US, Greece	SLE	NR	Blood	aIFN-α/β/ω	Multiplex bead-based assay	Y	191 (14%)	22 (12%)	NR	NR	12/10/7	6/5/3
Harris et al. (2020) [29]	US	SLE	NR	Blood, Urine	aIFN-α/β/ω	ELISA	Y	41 (8%)	28 (68%)	2 (5%)	NR	28/2/5	2/-/-
Mathian et al. (2022) [30]	France	SLE	NR	Blood	aIFN-α/β/ω	ELISA	Y	609 (6%)	71 (12%)	30 (5%)	NR	NR	18/12/15
Abers et al. (2021) [49]	Italy	COVID-19	82%	Blood	aIFN-α/β/ω	NR	Y	218 (75%)	NR	26 (12%)	24 (92%)	NR	22/1/21
Achleitner et al. (2023) [37]	Germany	COVID-19	100%	Blood	aIFN-α/β/ω	Luminex	Y	21 (86%)	3 (14%)	NR	NR	0/1/3	0/0/NR
Akbil et al. (2022) [22]	Germany, Switzerland	COVID-19	55% critical (no. of severe cases not reported)	Blood	aIFN-α/ω	Reverse ELISA	Y	430 (72%)	NR	18 (4%)	15 (83%)	NR	18/-/11
Antoli et al. (2025) [19]	Spain	COVID-19	56%	Blood	aIFN-α/ω	ELISA	Y	670 (53%)	92 (14%)	35 (5%)	NR	75/-/49	35/-/11
Aoki et al. (2024) [50]	Japan	COVID-19	100%	Blood	aIFN-α/β/ω	ELISA	Y	61 (85%)	10 (16%)	7 (11%)	7 (100%)	10/NR/NR	7/1/5
Arrestier et al. (2022) [51]	France	COVID-19	100%	Blood	NR	NR	Y	925 (70%)	NR	96 (10%)	75 (78%)	NR	74/12/71
Bastard et al. (2020) [9]	NR	COVID-19	100%	Blood	aIFN-α/β/ω	Multiplex particle-based flow cytometry, ELISA, Luminex	Y	987 (77%)	135 (14%)	101 (10%)	95 (94%)	94/19/90	88/2/65
Bastard et al. (2021) [52]	England, France, Italy, Russia, Scotland, Sweden and the US	COVID-19 in patients with autoimmune polyglandular syndrome type-1	68%	Blood	aIFN-α/β/ω	Multiplex particle-based assay	Y	21 (43%)	NR	21 (100%)	9 (43%)	NR	20/1/21
Bastard et al. (2021) [16]	38 countries	COVID-19	100%	Blood	aIFN-α/β/ω	Gyros	Y	3595 (73%)	NR	489 (14%)	NR	NR	360/NR/385
Bastard et al. (2023) [53]	France, Greece, North Macedonia, Turkey, Ukraine and the US	COVID-19 in patients with breakthrough critical infection despite recent vaccination	100%	Blood	aIFN-α/β/ω	RLBA	Y	42 (NR)	NR	10 (24%)	7 (70%)	NR	8/0/10
Bastard et al. (2024) [26]	Brazil, France, Italy, Morocco, Saudi Arabia, Spain, Peru, Turkey and Ukraine	COVID-19 in children aged 0–17	93%	Blood	aIFN-α/β/ω	Gyros	Y	183 (50%)	NR	19 (10%)	9 (47%)	NR	10/2/14
Bodansky et al. (2022) [27]	USA	COVID-19 in children <21 years of age	100%	Blood	aIFN-α	Radioligand binding assay	N	168 (49%)	1 (0.6%)	-	-	1/-/-	-
Boyarchuk et al. (2025) [28]	Ukraine	COVID-19 in children aged 1–17	8%	Blood	aIFN-α	ELISA	N	50 (58%)	2 (4%)	-	-	2/-/-	-
Busnadiego et al. (2022) [34]	Switzerland	COVID-19	100%	Blood. Tracheobronchial secretions	aIFN-α/β/ω	Bead-based multiplex immunoassay	Y	103 (78%)	12 (12%)	11 (11%)	9 (82%)	12/-/8	11/-/8
Chauvineau-Grenier et al. (2022) [17]	France	COVID-19	100%	Blood	aIFN-α/ω	Gyros	Y	139 (62%)	107 (77%)	11 (8%)	9 (81.8%)	NR	6/-/8
Cockx et al. (2024) [54]	Belgium	COVID-19	100%	Blood	aIFN-α	Luminex, ELISA	Y	52 (73%)	6 (12%)	4 (8%)	NR	6/-/-	4/-/-
Cognasse et al. (2022) [55]	France	COVID-19-studied convalescent plasma donations for antibodies	1.7%	Blood	aIFN-α/ω	Gyros	Y	766 (63%)	NR	14 (2%)	NR	NR	2/0/13
Covill et al. (2024) [24]	Sweden	Critical COVID-19 patients under 50 years of age	100%	Blood	aIFN-α	NR	Y	38 (76%)	NR	2 (5%)	NR	NR	2/-/-
Eto et al. (2022) [23]	Japan	COVID-19	65%	Blood	aIFN-α/ω	ELISA, ProQuantum immunoassay	Y	622 (71%)	19 (3%)	26 (4%)	NR	16/-/15	20/-/21
Framil et al. (2025) [33]	Spain	COVID-19	100%	Blood	aIFN-α/ω	ELISA	Y	275 (NR)	49 (18%)	26 (9%)	NR	NR	NR
Frasca et al. (2022) [56]	Italy	COVID-19	17% critical (no. of severe cases not reported)	Blood. Respiratory samples	aIFN-α/β/ω	ELISA	Y	360 (69%)	61 (17%)	13 (4%)	NR	27/37/NR	13/1/9
Gonçalves et al. (2021) [57]	France	COVID-19	100%	Blood	aIFN-α/β/ω	Commerical kit, ELISA	Y	84 (80%)	21 (25%)	15 (18%)	13 (87%)	21/1/10	15/-/10
Hansen et al. (2023) [35]	Denmark	Patients with long COVID	13%	Blood	aIFN-α/ω	ELISA	N	279 (25%)	5 (2%)	-	-	4/-/3	-
Joly et al. (2023) [58]	France	COVID-19	100%	Blood	aIFN-α/ω	Simoa kit	Y	140 (71%)	NR	27 (19%)	NR	NR	NR
Lamacchia et al. (2022) [59]	Italy	COVID-19	58%	Blood	aIFN-α	ELISA	N	65 (57%)	6 (9%)	-	-	6/-/-	-
Le Stang et al. (2024) [60]	France	COVID-19	100%	Blood	aIFN-α/β/ω	ELISA	Y	99 (77%)	NR	38 (38%)	31 (82%)	NR	NR
Lim et al. (2025) [25]	Singapore	COVID-19	15%	Blood	aIFN-α	ELISA	Y	122 (71%)	30 (25%)	5 (4%)	NR	30/-/-	5/-/-
Lopez et al. (2021) [61]	France	COVID-19	100%	Blood; Nasal mucosa	aIFN-α/ω	ELISA	Y	26 (73%)	NR	8 (31%)	NR	NR	NR
Manry et al. (2022) [32]	France	Fatal COVID-19 cases	100%	Blood	aIFN-α/ω	ELISA	Y	1121 (73%)	NR	305 (27%)	NR	NR	242/-/267
Peluso et al. (2022) [36]	USA	Patients with long COVID	22%	Blood	aIFN-α	Immunoprecipitation	N	215 (58%)	2 (1%)	-	-	2/-/-	-
Philippot et al. (2023) [21]	France, Netherlands	COVID-19	100%	BAL for all patients, Blood in 95 patients. Data based on BAL	aIFN-α/β/ω	Gyros	Y	415 (69%)	NR	54 (13%)—based on BAL samples	NR	NR	45/5/37
Pons et al. (2023) [62]	Peru	COVID-19	100%	Blood	aIFN-α	ELISA	N	54 (87%)	26 (48%)	-	-	26/-/-	-
Raadsen et al. (2021) [63]	Netherlands	COVID-19	100%	Blood, BAL	aIFN-α	ELISA	Y	135 (74%)	12 (9%)	12 (9%)	NR	12/-/-	12/-/-
Lafont Rapnouil et al. (2023) [64]	France	COVID-19	100%	Blood	aIFN-α/β/ω	NR	Y	231 (75%)	NR	30 (13%)	NR	NR	NR
Saheb Sharif-Askari F et al. (2023) [65]	Dubai	COVID-19	100%	Blood	aIFN-α/ω	ELISA	N	38 (89%)	20 (53%)	-	-	NR	-
Saheb Sharif-Askari N et al. (2024) [66]	United Arab Emirates	COVID-19	100%	Blood, Saliva	aIFN-α/ω	ELISA	Y	213 (80%)	42 (20%)	22 (10%)	NR	38/-/39	NR
Shi et al. (2024) [20]	China	COVID-19	100%	Blood	aIFN-α/ω	NR	Y	85 (61%)	NR	10 (12%)	NR	NR	9/-/8
Solanich et al. (2021) [18]	Spain	COVID-19	100%	Blood	aIFN-α/ω	ELISA	Y	275 (77%)	49 (18%)	26 (9%)	24 (92%)	41/-/30	25/-/22
Soltani-Zangbar et al. (2022) [67]	Iran	COVID-19	100%	Blood	aIFN-α	ELISA	N	50 (58%)	14 (28%)	-	-	14/-/-	-
Strunz et al. (2024) [68]	Sweden	COVID-19	100%	Blood	aIFN-α	NR	N	257 (76%)	11 (4%)	-	-	NR	-
Tiniakou et al. (2024) [69]	USA	COVID-19	100%	Blood	aIFN-α	ELISA	N	118 (56%)	10 (8%)	-	-	10/-/-	-
Troya et al. (2021) [70]	Spain	COVID-19	100%	Blood	aIFN-α/β/ω	ELISA	Y	47 (60%)	NR	5 (11%)	3 (60%)	NR	5/0/5
Van der Wijst et al. (2021) [71]	USA	COVID-19	100%	Blood	aIFN-α	RLBA	Y	128 (NR)	11 (9%)	10 (8%)	NR	11/-/-	10/-/-
Vanker et al. (2023) [72]	Estonia, Denmark, France and UK	COVID-19	50%	Blood	aIFN-α	LIPS	Y	1002 (47%)	55 (5%)	NR	NR	55/-/-	NR
Von Stemann et al. (2024) [73]	Denmark	COVID-19	NR	Blood	aIFN-α/β	Luminex	N	97 (60%)	14 (14%)	-	-	14/13/-	-

Note. ALBIA, addressable laser bead immunoassay; ELISA, enzyme-linked immunosorbent assay; LIPS, luciferase immunoprecipitation system; RLBA, radioligand binding assay; NR, not reported; “-“ indicates not tested or not applicable
